# Effects of Lichenic Extracts *(Hypogymnia physodes, Ramalina polymorpha and Usnea florida)* on Human Blood Cells: Cytogenetic and Biochemical Study

**Published:** 2012

**Authors:** Hasan Türkez, Elanur Aydın, Ali Aslan

**Affiliations:** a ***Department of Molecular Biology and Genetics, Faculty of Science, Erzurum Technical University, Erzurum, Turkey.***; b ***Atatürk University, Kazım Karabekir Education Faculty, Department of Biology, 25240, Erzurum, Turkey.***

**Keywords:** Chromosomal aberrations, *Hypogymnia physodes*, Micronucleus assay, *Ramalina polymorpha*, Total antioxidant capacity, Total oxidant status, *Usnea florida*

## Abstract

Several lichen species have been used for medicinal purposes throughout the ages, and they were reported to be effective in the treatment of different disorders including tuberculosis, hemorrhoids, ulcer, dysentery and cancer. It is revealed that they may be easily accessible sources of natural drugs that could be used as a possible food supplement or in pharmaceutical industry after their safety evaluations. However, so far, the nature and/or biological roles of plenty of lichenes have not been elucidated exactly. The aim of this study was to investigate the genetic and oxidative effects of water extracts of three different lichen species; *Hypogymnia physodes, Ramalina polymorpha *and *Usnea florida *in cultured human blood cells (n = 5) for the first time. All lichen species were collected from the Erzurum and Artvin provinces (in Turkey) during August 2010. The lichen extracts were added into culture tubes at various concentrations (0 to 2000 mg/L). Chromosome aberrations (CA) and micronucleus (MN) tests were used for genotoxic influences estimation. In addition, biochemical parameters (total antioxidant capacity (TAC) and total oxidative stress (TOS)) were examined to determine oxidative effects. In our in-vitro test systems, it was observed that all tested lichen extracts had no mutagenic effects on human lymphocytes. Furthermore, these extracts exhibited antioxidant properties due to the type of lichen species added to the cultures. In conclusion, these lichens can be a new resource of therapeutics as recognized in this study with their non-mutagenic and antioxidant features.

## Introduction

Lichens are composite organisms consisting of a symbiotic association of a fungus (the mycobiont) with a photosynthetic partner (the photobiont), usually either a green algae or cyanobacterium (1). Lichens were effective in the treatment of diseases such as hemorrhoids, bronchitis, dysentery, and tuberculosis (2). Again, lichen species have been used as stomachic, antidiabetic, and hemostatic drug (3). Some recent studies have also revealed that secondary metabolites from lichens induce apoptosis in colon (4, 5) and prostate (6) cancers. Lichens have long been investigated for biological activities; mainly antimicrobial but also antitumor, antiviral, allergenic, plant growth inhibitory, antiherbivore, and enzyme inhibitory (7), more recently, antioxidant and anti-inflammatory activities (8-10).

Antioxidants could inhibit or delay the oxidation process by blocking the initiation or propagation of oxidizing chain reactions. A variety of synthetic antioxidants like butylated hydroxyanisole, butylated hydroxytoluene, and tert-butylhydroquinone are commonly used within the food industry although restrictions on the use of synthetic antioxidants are being imposed because of their toxicity (11-13). Much attention has recently been focused on the development of safe and effective antioxidants (14), because toxic free radicals play a role in the etiology of many disorders including neurodegenerative and cardovascular diseases (15, 16), diabetes (18) and certain cancers (19). Therefore, the development and utilization of more effective and less harmful antioxidants of natural origins are reported to be very desirable (13).

Lichen species are very common in Turkey. It is pointed that they may be easily accessible sources of natural drugs that could be used as a possible food supplement or in pharmaceutical industry (20). In this investigation, it was aimed to describe the cytogenetic and oxidative effects of three lichen species; *Hypogymnia physodes, Ramalina polymorpha *and *Usnea florida *on cultured human blood cells for utilization as a possible food supplement or within the pharmaceutical industry. And, safety screening of these lichen extracts could also serve to explore new natural therapeutics. With this aim, not only CA frequencies but also MN formations have been established as related to the dose of aqueous extracts of lichenes on human lymphocytes. In addition, important oxidative parameters, TAC and TOS were used to monitor their antioxidant or pro-oxidant activities *in-vitro*.

## Experimental

*Plant materials*

Lichen species, *Hypogymnia physodes *(L.) Nyl.*, Ramalina polymorpha *(Lilj.) Ach. and *Usnea florida *(L.) Wigg. ex Web. em Clerc. were collected from the Erzurum and Artvin provinces during August 2010. After drying at the room temperature a stereo microscope (since it produces a three-dimensional visualization of the sample being examined) and the usual spot tests were used in the identification of the samples with the reference books (20-23). The specimens are stored in the herbarium of Kazım Karabekir, Faculty of Education, Atatürk University, Erzurum.

*Extraction*

For water extraction out of lichenes, 20 g sample was mixed with 400 mL distillated and boiling water using magnetic stirrer for 15 min. Then the extracts were filtered over Whatmann No. 1 paper.

*Experimental design*

Whole heparinized human blood from five healthy non-smoking female donors between the ages 27 and 28 with no history of exposure to any genotoxic agent was used in our experiments. Questionnaires were obtained for each blood donor to evaluate exposure history, and in addition, informed consent forms were signed by each donor. In all the volunteers involved in this study, hematological and biochemical parameters were analyzed and no pathology was detected. A various concentrations (0, 1, 5, 10, 25, 50, 100, 200, 250, 500, 1000 and 2000 mg/L) of aqueous extracts of lichenes were tested in blood cultures. CA and MN rates were assessed in peripheral lymphocytes. Experiments conformed to the guidelines of the World Medical Assembly (Declaration of Helsinki) (24). The cultures without extracts were studied as control^-^ group. Mitomycin C (10^-7^M) was used as the positive control in CA and MN assays. Likewise, ascorbic acid (10 μM) and hydrogen peroxide (25 μM) were also used as the positive controls in TAC and TOS analysis, respectively.

*CA assay*

Human peripheral blood lymphocyte cultures were set up according to a slight modification of the protocol described by Evans and O’Riordan (25). A 0.5 mL aliquot of heparinized blood was cultured in 6 mL of culture medium (Chromosome Medium B; Biochrom®, Berlin) with 5 mg/mL of phytohemagglutinin (Biochrom®). The cultures were incubated in complete darkness for 72 h at 37ºC. Two hours prior to harvesting, 0.1 mL of colchicine (0.2 mg/mL, Sigma®) was added to the culture flask. Hypotonic treatment and fixation were performed. To prepare slides, 3–5 drops of the fixed cell suspension were dropped on a clean slide and air-dried. The slides were stained in 3% Giemsa solution in phosphate buffer (pH 6.8) for 15 min. For each treatment, 30 well-spread metaphases were analyzed to detect the presence of chromosomal aberrations. Criteria to classify the different types of aberrations (chromatid or chromosome gap and chromatid or chromosome break) were in accordance with the recommendation of EHC (Environmental Health Criteria) 46 for environmental monitoring of human populations (26).

*MN assay*

**Figure 1:**
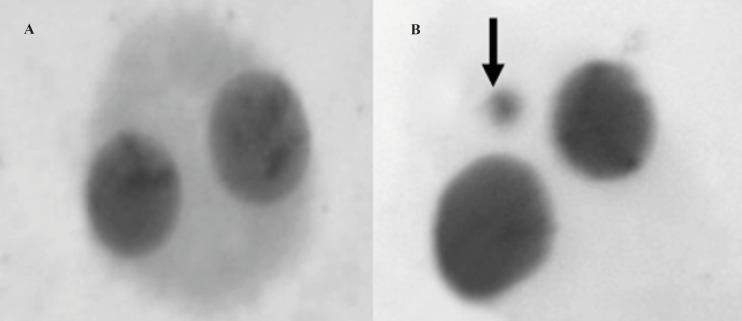
A sample binucleated cells (A) and micronucleus (arrow) formation (B) in lymphocyte cells

The micronucleus test was performed by adding cytochalasin B (Sigma®; final concentration of 6 μg/mL) after 44 h of culture. At the end of the 72-h incubation period, the lymphocytes were fixed with ice-cold methanol: acetic acid (1:1). The fixed cells were put directly on slides using a cytospin and stained with Giemsa. All slides were coded before scoring. The criteria for scoring micronuclei were as described by Fenech (27). A sample binucleated lymphocyte cells and micronucleus formation was shown in Figure 1A and B, respectively. At least 1000 binucleated lymphocytes were examined per concentration for the presence of one, two, or more micronuclei.

*TAC and TOS analysis*

The automated total antioxidant capacity and total oxidant status assays were carried out in plasma samples obtained from blood cultures for 2 h by commercially available kits (Rel Assay Diagnostics®, Turkey) (27).

*Statistical analysis*

Statistical analysis was performed using SPSS software (version 13.0, SPSS, Chicago, IL, USA). The Duncan’s was used to determine whether any treatment significantly differed from controls or each other. Statistical decisions were made with a significance level of 0.05.

## Results and Discussion

Tables 1, 2, and 3 show the genetic and biochemical data obtained with various concentrations of *Hypogymnia physodes, Ramalina polymorpha *and *Usnea florida*, on cultured blood cells, respectively. All the lichen extracts at tested concentrations did not induce significant (p < 0,05) number of CAs and MNs. However, the Mitomycin C applied culture (positive control) showed about three fold increases of both parameters as compared to control^- ^group.

**Table 1:** The genetic and biochemical effects of aqueous extracts of *Hypogymnia physodes in-vitro.*

**Treatments**	**TAC (mmol Trolox Equiv./L)**	**TOS (μmol H**_2_ **O**_2_ **Equiv./L)**	**MN/1000 cell**	**CA/cell**
Control^-^	6.24 ± 0.62^b^	11.58 ± 2.64^a^	3.18 ± 0.72^a^	0.20 ± 0.02^a^
Control^+^	13.71 ± 0.94^d^	39.25 ± 4.63^c^	8.26 ± 1.18^b^	0.72 ± 0.09^b^
1 mg/L	6.25 ± 0.43^b^	11.27 ± 2.76^a^	3.35 ± 0.48^a^	0.20 ± 0.03^a^
5 mg/L	6.27 ± 0.42^b^	11.02 ± 2.54^a^	3.40 ± 0.55^a^	0.21 ± 0.02^a^
10 mg/L	6.25 ± 0.39^b^	11.01 ± 3.01^a^	3.37 ± 0.62^a^	0.23 ± 0.02^a^
25 mg/L	6.26 ± 0.48^b^	11.60 ± 2.74^a^	3.39 ± 0.64^a^	0.21 ± 0.03^a^
50 mg/L	7.22 ± 0.41^c^	11.63 ± 3.02^a^	3.36 ± 0.59^a^	0.22 ± 0.01^a^
100 mg/L	6.27 ± 0.38^b^	11.62 ± 2.94^a^	3.34 ± 0.63^a^	0.18 ± 0.03^a^
200 mg/L	6.26 ± 0.45^b^	11.64 ± 2.85^a^	3.37 ± 0.61^a^	0.21 ± 0.02^a^
250 mg/L	6.28 ± 0.44^b^	12.03 ± 3.10^b^	3.32 ± 0.58^a^	0.18 ± 0.03^a^
500 mg/L	6.27 ± 0.47^b^	12.27 ± 3.04^b^	3.36 ± 0.51^a^	0.22 ± 0.04^a^
1000 mg/L	5.59 ± 0.43^a^	13.35 ± 3.12^b^	-	-
2000 mg/L	5.12 ± 0.46^a^	14.02 ± 3.40^b^	-	-

Values are presented as mean±S.D.; n = 5, means shown by the same letter are not significantly different from each other at a level of 5%, using Duncan’s test. For example, the mean shown by the letter b is different from a, c and d means, but not different from b mean. In other words, the means having the different letters are different from each other

**Table 2:** The genetic and biochemical effects of aqueous extracts of *Ramalina polymorpha in-vitro.*

**Treatments**	**TAC (mmol Trolox Equiv./L)**	**TOS (μmol H**_2_ **O**_2_ **Equiv./L)**	**MN/1000 cell**	**CA/cell**
Control^-^	6.24 ± 0.62^b^	11.58 ± 2.64^a^	3.18 ± 0.72^a^	0.20 ± 0.02^a^
Control^+^	13.71 ± 0.94^d^	39.25 ± 4.63^c^	8.26 ± 1.18^b^	0.72 ± 0.09^b^
1 mg/L	6.22 ± 0.56^b^	11.54 ± 2.83^a^	3.22 ± 0.45^a^	0.21 ± 0.02^a^
5 mg/L	6.29 ± 0.61^b^	11.63 ± 3.05^a^	3.25 ± 0.49^a^	0.24 ± 0.03^a^
10 mg/L	6.31 ± 0.49^b^	11.49 ± 2.75^a^	3.27 ± 0.43^a^	0.22 ± 0.02^a^
25 mg/L	6.69 ± 0.54^b^	11.13 ± 3.18^a^	3.35 ± 0.41^a^	0.20 ± 0.02^a^
50 mg/L	9.29 ± 0.79^c^	11.56 ± 2.86^a^	3.29 ± 0.48^a^	0.21 ± 0.03^a^
100 mg/L	6.58 ± 0.62^b^	11.49 ± 2.74^a^	3.32 ± 0.46^a^	0.23 ± 0.02^a^
200 mg/L	6.63 ± 0.67^b^	11.47 ± 3.07^a^	3.37 ± 0.51^a^	0.21 ± 0.02^a^
250 mg/L	6.71 ± 0.48^b^	11.72 ± 2.93^a^	3.29 ± 0.58^a^	0.20 ± 0.03^a^
500 mg/L	6.30 ± 0.51^b^	11.74 ± 3.16^a^	3.36 ± 0.54^a^	0.22 ± 0.02^a^
1000 mg/L	6.09 ± 0.45^b^	11.81 ± 2.88^a^	3.24 ± 0.61^a^	0.21 ± 0.02^a^
2000 mg/L	5.71 ± 0.47^a^	11.86 ± 3.03^a^	-	-

Values are presented as mean±S.D.; n=5, means shown by the same letter are not significantly different from each other at a level of 5%, using Duncan’s test. For example, the mean shown by the letter b is different from a, c and d means, but not different from b mean. In other words, the means having the different letters are different from each other

**Table 3:** The genetic and biochemical effects of aqueous extracts of *Usnea florida in-vitro*

**Treatments**	**TAC (mmol Trolox Equiv./L)**	**TOS (μmol H**_2_ **O**_2_ **Equiv./L)**	**MN/1000 cell**	**CA/cell**
Control^-^	6.24 ± 0.62^b^	11.58 ± 2.64^a^	3.18 ± 0.72^a^	0.20 ± 0.02^a^
Control^+^	13.71 ± 0.94^d^	39.25 ± 4.63^c^	8.26 ± 1.18^b^	0.72 ± 0.09^b^
1 mg/L	6.27 ± 0.53^b^	11.97 ± 2.14^a^	3.43 ± 0.63^a^	0.22 ± 0.03^a^
5 mg/L	6.30 ± 0.62^b^	11.73 ± 3.13^a^	3.27 ± 0.34^a^	0.20 ± 0.04^a^
10 mg/L	6.25 ± 0.57^b^	11.58 ± 2.42^a^	3.18 ± 0.22^a^	0.20 ± 0.02^a^
25 mg/L	6.38 ± 0.61^b^	11.33 ± 3.07^a^	2.87 ± 0.39^a^	0.20 ± 0.02^a^
50 mg/L	6.69 ± 0.67^b^	11.56 ± 2.88^a^	3.32 ± 0.55^a^	0.18 ± 0.03^a^
100 mg/L	7.33 ± 0.49^c^	11.14 ± 2.43^a^	3.23 ± 0.35^a^	0.15 ± 0.03^a^
200 mg/L	6.51 ± 0.55^b^	11.63 ± 3.17^a^	3.25 ± 0.38^a^	0.22 ± 0.03^a^
250 mg/L	6.35 ± 0.57^b^	11.72 ± 2.43^a^	3.39 ± 0.57^a^	0.24 ± 0.02^a^
500 mg/L	6.18 ± 0.59^b^	11.82 ± 2.77^a^	3.41 ± 0.69^a^	0.24 ± 0.02^a^
1000 mg/L	5.43 ± 0.43^a^	11.88 ± 3.14^a^	-	-
2000 mg/L	5.22 ± 0.51^a^	12.76 ± 3.27^b^	-	-

Values are presented as mean±S.D.; n=5, means shown by the same letter are not significantly different from each other at a level of 5%, using Duncan’s test. For example, the mean shown by the letter b is different from a, c and d means, but not different from b mean. In other words, the means having the different letters are different from each other

In the present study, it was established that the extracts of *H. physodes, R. polymorpha *and *U. florida *lichen species were non-genotoxic. The results obtained through the preseht stugy did not indicate any significant increases in the ratios of the CAs and MNs in lymphocytes exposed to lichen extracts as compared to control values. In fact, CA test is regarded as a very important and useful indicator of exposure to biological and chemical agents (29). MN assay provides a measure of both chromosome breakage and chromosome loss or non-disjunction in clastogenic and aneugenic events, respectively (30). And damaged DNA can lead to aneuploidy and/or chromosomal instability, which is believed to be major contributor to tumor progression (31). Our findings are in accordance with the previous reports. Koparal *et al. *(32) investigated cytotoxic and genotoxic activities of the lichen *Ramalina farinacea *and the lichen *Cladonia foliacea *and suggested that usnic acid (a main component of lichens) was non-genotoxic shown by the absence of MN induction in human lymphocytes. Again, Zeytinoglu *et al. *(33) investigated the genotoxic/antigenotoxic activities of the extract from lichen *Cetraria aculeata *in TA98 and TA100 strains of Salmonella typhimurium in the presence or absence of metabolic activity and in human lymphocytes. They have reported that the lichen extract was not mutagenic in all systems. Again, the genotoxic effects of the water extracts of *Pseudevernia furfuracea, Dermotocarpon intestiniforme, Ramalina capitata, Parmelia pulla *and *Rhizoplaca melanophthalma *lichens were ascertained by sister-chromatid exchange (SCE) and MN tests in human whole blood cultures. According to results of this study, it was established that these lichen extracts had also no genotoxic effect (34). The separated components of Chinese lichen extract such as AMH-C, AMH-D and AMH-E were determined as non genotoxic in Ames test (35). Turkez *et al. *(10) studied the effects of methanol, acetone, n-hexane and ether extracts obtained from the lichen, *Pseudovernia furfuracea*, on genotoxicity in cultured human blood cells by SCE and and MN tests. The researchers observed that *P. furfuracea *extracts exhibited non-mutagenic properties in both test systems.

Different concentrations of *H. physodes *and *R. polymorpha *(50 mg/L) and *U. florida *(100 mg/L) caused significant increases of TAC level when compared to control^- ^value. In contrast, *H. physodes *(at concentrations of 1000 and 2000 mg/L), *R. polymorpha *(at concentration of 2000 mg/L) and *U. florida *(at concentrations of 1000 and 2000 mg/L) caused significant decreases of TAC level. As shown from the results presented in Tables 1, 2 and 3, the TOS levels increased at higher concentrations of *H. physodes *(250, 500, 1000 and 2000 mg/L) and *U. florida *(2000 mg/L). However, *R. Polymorpha *did not cause any significant increases of TOS levels. Besides, the cultures found to be sterile at concentartions of 1000, 2000 and 1000 mg/L for *H. Physodes, R. polymorpha *and *U. florida*, respectively.

The results of the present study reveal that treatment with aqueous extracts of *H. physodes, R. polymorpha *and *U. florida *lichen species provide antioxidant effects at different degree. Similarly to our findings, various reports, especially published in last ten years, indicated the antioxidant properties of several lichen species. According to the previous studies, the extracts of *Cladonia clathrata *(8), *Pseudovernia furfuracea *(10), *Xanthoparmelia spp*. (36), *Lethariella sernanderi, L. cashmeriana*, and *L. sinensis *(37), *Lobaria pulmonaria *(38), *Usnea ghattensis *(39), *Usnea longissima *(40), Graphidaceae (41), *Lethariella canariensis *(42), *Cetraria islandica *(19), *Parmelia caperata *and *P. soredians *(43), *Dermatocarpon miniatum *(44), *Parmotrema stuppeum *(45) were found to have antioxidant properties.

The results of the present study also indicated that the aqueous extracts of *H. physodes *and *U. florida *lichen species caused sterility of human blood cultures for 72 h at concentration of 2000 mg/L. Similarly, *R. polymorpha *caused sterility at concentration of 1000 mg/L. On the other hand, *H. physodes *(at concentrations of 250, 500, 1000 and 2000 mg/L) and *U. florida *(at concentration of 2000 mg/L) caused significant increases of TOS levels at increasing doses. However, *R. Polymorpha *did not cause significant increases of TOS levels. Hence, the cytotoxic effects of this lichen species could be, at least in part, attributed to oxidative stress-induced by high lichenic contents. The multidisciplinary toxicity studies can be of help here as well. But cytotoxic actions of these lichens at higher concentrations were also could be due to initation of cell death, inhibition of functional enzymes, antimitochondrial actions, binding to plasma membrane and changes in osmotic potential. Therefore, further investigations are neccessary to find out the definite mechanisms in lichen toxicity.

In conclusion, our results clearly indicated that the water extracts of *H. physodes, R. polymorpha *and *U. florida *lichen species collected from Erzurum and Artvin provinces had no mutagenic effects on human lymphocytes. Furthermore, these extracts exhibited antioxidant properties due to the applied dose and the type of lichen species added to the cultures. On the other hand, the extracts caused sterility of cultures due to oxidative stress at higher concentrations above than 1000 mg/L. Generally speaking, our results have indicated that several North East Anatolian lichens have the potential of being utilized as novel bioresources for naturally occurring antioxidant therapies.
